# The Role of Dorsal Anterior Cingulate Cortex in the Regulation of Craving by Reappraisal in Smokers

**DOI:** 10.1371/journal.pone.0043598

**Published:** 2012-08-22

**Authors:** Li-Yan Zhao, Jie Tian, Wei Wang, Wei Qin, Jie Shi, Qiang Li, Kai Yuan, Ming-Hao Dong, Wei-Chuang Yang, Ya-Rong Wang, Li-Li Sun, Lin Lu

**Affiliations:** 1 National Institute on Drug Dependence, Peking University, Beijing, China; 2 Xidian University, Xian, China; 3 Tangdu Hospital, The Fourth Military Medical University, Xian, China; University College London, United Kingdom

## Abstract

**Rationale and Objective:**

Drug cues can induce craving for drugs of abuse. Dysfunctional regulation of emotion and motivation regarding rewarding objects appears to be an integral part of addiction. It has been found that cognitive strategies decreased the intensity of craving in addicts. Reappraisal strategy is a type of cognitive strategy that requires participants to reinterpret the meaning of an emotional situation. In addition, studies have found that activation of the dorsal anterior cingulate cortex (dACC) is associated with the selection and application of cognitive reappraisal. In present study, we sought to determine whether such cognitive regulation engages the dACC and improves inhibition of craving in smokers.

**Methods:**

Sixteen smokers underwent functional magnetic resonance imaging (fMRI) during performance of a cigarette reward-conditioning procedure with cognitive reappraisal. We focused our analyses on the dACC as a key structure of cognitive control of craving. Cue induced craving under different conditions was obtained. Correlational analysis between the functional response in the dACC and the subjective craving was performed.

**Results:**

We found that using a cognitive reappraisal was successful in decreasing the conditioned craving. Right dACC (BA 24/32) engaged in the cognitive reappraisal. In addition, the individual’s subjective craving was negatively correlated with the right dACC activation.

**Conclusions:**

These findings suggest that the dACC are important substrates of Inhibition of cue induced craving in smokers. Cognitive regulation by cognitive reappraisal may help addicted individuals avoid the anticipated situations where they are exposed to conditioned cues.

## Introduction

The presence of cigarette-related cues has been implicated as a precipitating factor in inducing craving and causes many relapse episodes [1]. Craving for cigarette is an important contributor to nicotine addiction, and regulation of craving are effectively in reducing rates of relapse. Thus, reducing reactivity to cigarette-related cues appears to be a promising strategy for improving the success of quit attempts [2]. Reappraisal strategies have been shown to successfully modulate the subjective emotional state and activation in brain areas relevant for emotional processing including the dorsal anterior cigulate cortex (dACC) [3]. Thus, It is vitally important to determine whether reappraisal strategy influence reward processing at the neural level. This clinical approach has recently been replicated in a laboratory model of cognitive strategies to modulate the intensity of craving. We further examined this laboratory model using functional magnetic resonance imaging (fMRI) to understand the neural mechanisms that underlie this cognitive strategy for reducing cigarette-related cue induced craving.

In the present study, reappraisal was used as the cognitive strategy to regulate the response induced by cigarette-related cues. Reappraisal strategy requires participants to reinterpret the meaning of an emotional situation. Studies have found that activation of the ACC is associated with the selection and application of reappraisal strategies. Prior research has demonstrated activation of the dACC during active reappraisal and modulation of emotional responses to cues [4], [5], [6], [7]. Mental effort has also been linked to activation of the dACC [8]. In addition, the functional connectivity within the circuit of cognitive control (between ACC and PFC) was abnormal in drug abusers [9], [10]. Based on these studies and the imaging studies cited above, we hypothesized that the dACC is activated in response to cognitive strategies that regulate craving during cigarette-related cue exposure in smokers.

To investigate the effect of a cognitive reappraisal on cigarette cue-induced craving in nicotine addicts, the present study used a reward conditioning paradigm with instruction and partial reinforcement [11]. We predicted that the reappraisal strategy would reduce cigarette cue-induced craving and be accompanied by increased metabolic activity in the dACC, which is a brain region critical for exerting cognitive behavioral control [12].

## Methods

### Participants

The subjects provided written informed consent, and the study was approved by the Peking University Research Ethics Board. Participants were recruited using posted advertisements or by word of mouth and were compensated $20 for study participation. The participants were screened for psychiatric disorders using an interview based on the Structure Clinical Interview for the Diagnostic and Statistical Manual Disorders (SCID for DSM-IV) by the clinician. The inclusion criteria were the following: (1) men who met the *Diagnostic and Statistical Manual of Mental Disorders*, 4th edition (DSM-IV), criteria for current nicotine dependence, (2) no history of any neurological or psychiatric disorder and no other drug dependence, (3) at least 15 cigarettes smoked per day for at least 5 years, (4) right-handed, (5) 25 to 50 years old. The exclusion criteria were the following: (1) any lifetime DSM-IV Axis I disorder other than nicotine dependence, (2) use of nicotine replacement therapy (NRT) or another smoking cessation treatment, (3) current use of medications that could alter brain function, and (4) history of head trauma. Smokers were instructed to cease cigarette or tobacco use overnight, approximately 12 h before the study. Their compliance was confirmed by breath carbon monoxide (CO) levels below 10 ppm on a sample of expired air (Micro Smokerlyzer MP011095, Bedfont Scientific, England) [13].

Twenty right-handed individuals were recruited. Two subjects were excluded due to data quality issues (primarily head motion) and two subject failed to follow the indicated cognitive reappraisal. Therefore, the four participants were excluded before analysis. The analysis cohort included 16 adult male smokers between 27 and 46 years of age (41.3±5.2 years, mean ± SD). The Intelligence Quotient (IQ; estimated using the Wechsler Intelligence Scale) was 102±6 (90 to 110), and the average years of education were 12.2±2.5 years (8 to 15). They had a mean lifetime history of regular smoking of 21.0±5.8 years (13 to 30) and smoked an average 20.7±5.6 cigarettes per day (9 to 30). Their breath CO level at screening was 25.9±4.6 ppm (16 to 38) and was 5.6±3.3 ppm (3 to 9) after ceasing cigarette use overnight. The mean score on the FTND questionnaire was 7.12±1.8 (5 to 10), indicating heavy nicotine dependence. The average BDI and HAM-A scores were 2.2±1.8 (0 to 8) and 1.2±1.8 (0 to 4) respectively.

### Task

**Figure 1:**
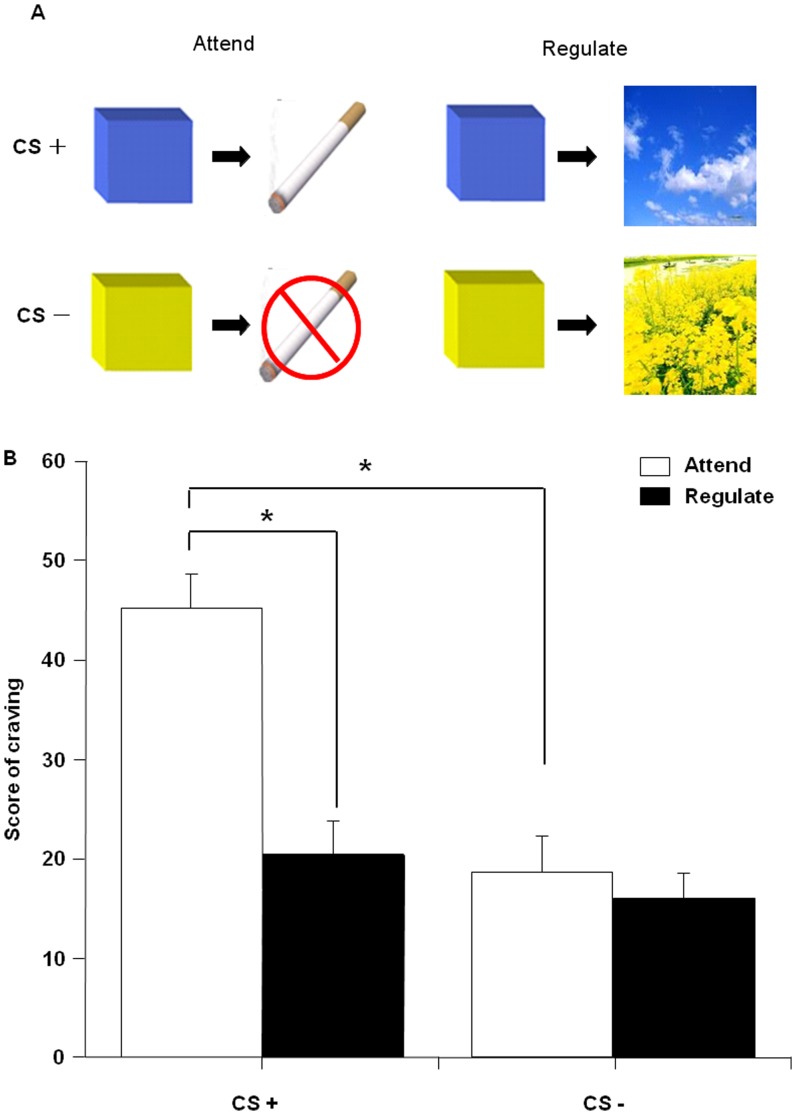
Description of the task and craving scores under four different trial types. (A) 1. When Attend instruction preceded a CS+ trial (e.g., blue square), the participants were taught to think about the possibility of winning a pack of cigarettes. 2. When Attend was paired with the CS– (e.g., yellow square), the participants were taught to think about the fact that no cigarettes are gained. 3 and 4. When the instruction Regulate appeared on the screen, participants were taught to conjure a soothing image from nature regardless of the color of the square (e.g., blue = CS+ or yellow = CS−). (B) Craving scores from 16 participants under different trial types (Attend CS+, Attend CS−, Regulate CS+, and Regulate CS−). Data are expressed as mean ± SEM. *p<0.05.

We used a reward conditioning paradigm with instruction and partial reinforcement [11]. The paradigm includes two conditioned stimuli (CSs): blue and yellow squares that are either paired (CS+) or unpaired (CS−) with a potential cigarette reward (a picture of a packet of cigarettes that indicated the subjects got a package of cigarettes). The CS+ was paired with cigarette reward on a 28.6% partial reinforcement schedule. Since we were only interested in the response induced by the CS, the partial reinforcement paradigm was used. Therefore, all trials on which a shock was delivered were excluded from analysis. Prior to CS presentation, participants were presented with a cue, a single word instruction that told participants to either Attend or Regulate the stimulus. Participants were explicitly told that when the instruction Regulate appears on the screen, they were instructed to conjure a soothing image from nature prompted by the color of the square (e.g. a blue square, participants might think of the sky and a yellow square, they might think of a field of cole flowers) and when the instruction Attend appears on the screen, the participants were instructed to think of their feelings and react normally (e.g. “I may get a pack of cigarette”, “I am craving for a cigarette” or “I will not get a pack of cigarette”). Additionally, participants were told that regardless of the instruction, the CS+ always indicated the possibility of winning a pack of cigarettes, independent of the Attend or Regulate condition. These two instruction words were presented on a black background for 2 s, followed by the CS presentation for 4 s, and followed by an interstimulus interval of 12 s, during which time the participant looked at a fixation point. The probabilistic cigarette picture was presented for 500 ms and co-terminated with the CS+ (Fig. 1A). If the CS+ was not followed by the cigarette picture, only the CS+ was presented for 4 s. Participants were told that they would win a pack of cigarettes with each cigarette picture presentation and accumulate the cigarettes throughout the experiment. The conditions were counterbalanced across participants. The subjects completed 72 trials separated into three blocks of 24 trials each. Within these blocks were 15 trials for each condition (Attend CS+, Attend CS–, Regulate CS+, and Regulate CS–) and an additional 12 trials that combined with the cigarette reward (CS–cigarette reward trials, six each for the Attend and Regulate conditions).

To avoid the effects of motor learning on behavioral measures, participants were asked to rate their cigarette craving outside the MRI scanner after scanning to evaluate validity of cognitive reappraisal. All images presented inside the scanner were presented a second time on a computer screen after scanning. Craving was assessed after each CS with the QSU that was required to be finished in 30 s. Craving scores during the four conditions were compared using a repeated-measures analysis of variance (ANOVA) of trial type (Attend trials, Regulate trials) and stimulus (CS+, CS–).

Before entering the scanner, the participants completed a set of practice trials. They were asked to perform the following reappraisal strategies during the four different conditions (i.e., Attend CS+, Attend CS–, Regulate CS+, and Regulate CS–). The research staff asked the participants to verbalize what they were thinking during each condition to ensure that they were following the indicated reappraisal strategy. Prior to the scanning session, therefore, participants were aware of the contingencies (e.g., blue square predicts a potential cigarette gain) and were well-practiced in the instructions and cognitive reappraisal strategies. After the scanning, the participants were also asked to verbalize what they were thinking during each condition and were excluded if they did not follow the indicated cognitive reappraisal.

### Clinical Assessment

On the experimental day, the Hamilton Anxiety Rating Scale (HAM-A) [14], Beck Depression Inventory (BDI) [15], Fagerström Test for Nicotine Dependence (FTND) [16], and Questionnaire of Smoking Urges (QSU) [17] were administered to all subjects. The HAM-A is a rating scale that measures the severity of anxiety symptoms. The scale consists of 14 items, each defined by a series of symptoms, and measures both psychic anxiety and somatic anxiety. The BDI is a rating scale that measures the severity of depression symptoms. The scale consists of 21 items. Items 1 to 13 assess symptoms that are psychological in nature, and items 14 to 21 assess physical symptoms. Subjects were then asked to complete a set of practice trials and verbalize what they were thinking during each condition to ensure that they understood the contingencies and were able to perform these reappraisal strategies. The QSU consists of 10 items with analog ratings (from 1 [not at all] to 7 [most ever]). Items on this scale included the desire to smoke freely, urge and craving for a cigarette, desire to smoke as soon as possible, degree to which a cigarette would be pleasant, and extent to which the subject misses a cigarette.

### Image Acquisition

A 3T GE Signa scanner and a standard head coil were used for brain imaging at Tangdu Hospital, Xi’an City. Anatomical images were acquired using a T1-weighted protocol (256×256 matrix, 166 transverse slices with 1 mm each). Functional images were collected using a gradient echo EPI sequence (64×64 matrix, 32 transverse slices with 4 mm, repetition time [TR] = 2000 ms, echo time [TE] = 30 ms, field of view [FOV] = 256×256 mm^2^, flip angle = 90°, voxel size 4×4×4 mm^3^). Scanning was divided into three runs that corresponded to three blocks of stimulus presentations.

**Table 1:** Regions activated by a contrast of Attend CS+ versus Regulate CS+ and vice versa (p<0.001).

Brain regions	Brodman’s Area (BA)	Talairach Coordinates (x, y, z)	Number of Voxels
**Regulate CS+ versus Attend CS+ Trials**			
**Right dACC**	BA24/32	8, 32, 24	48
**Right Cuneus**	BA19	8, −84, 36	68
**Right Inferior Parietal Lobule**	BA40	30, −38, 57	10
**Left Paracentral Lobule**		−8, −40, 57	64
**Right Paracentral Lobule**		8, −28, 60	34
**Right Cerebellum**		38, −52, −24	52
**Attend CS+ versus Regulate CS+ Trials**			
**Right vlPFC**	BA44/45		

### Image Analysis

Image preprocessing and statistical analysis were carried out with SPM5 (http://www.fil.ion.ucl.ac.uk/spm). The data was initially corrected for rigid body motion and slice timing. A threshold of 1 degree rotation in any of the axes and 1-mm displacement was used as criteria for acceptable motion. The realigned functional images were then spatially normalized to the EPI template in SPM5 in Montreal Neurological Institute (MNI) space http://imaging.mrc-cbu.cam.ac.uk/imaging/Templates) and re-sampled to 2 mm×2 mm×2 mm voxels. Further, spatial smoothing was performed using an isotropic Gaussian filter with 8 mm full width at half maximum (FWHM) to decrease spatial noise.

Subject-level statistical analyses used FSL’s Improved Linear Model with local autocorrelation correction [18]. The four condition events (Attend CS+, Attend CS−, Regulate CS+, and Regulate CS−) were separately modeled using a canonical hemodynamic response function indicating onset and duration (4 seconds) of each stimulus epoch. Motion correction parameters were included as nuisance covariates and the rest condition (fixation point) was treated as baseline. Group data were analyzed using a random effects model to determine blood oxygenation dependent signal (BOLD) for task-specific events. There were four predictors of interest (Attend CS+, Attend CS–, Regulate CS+, and Regulate CS–). Trials where the cigarette picture was delivered (CS–cigarette reward trials) were excluded from analysis. Statistical maps of interest were created using a threshold of p<0.001 (uncorrected). The primary contrast of interest was the differential response between Attend CS+ and Regulate CS+, which directly investigated the effect of using emotion regulation to diminish craving. To further investigate the effects of reappraisal strategy on craving, we examined BOLD responses in a priori regions of interest (ROI) based on previous studies of emotion regulation [19]. The ROI was the dACC that was previously implicated in emotion regulation. For the ROI, the peak differential BOLD response was identified in the group analysis (Attend versus Regulate CS+), and a cube was drawn around it (3 mm^3^). For the priori ROI, a multi-study GLM was performed on each individual participant, allowing for acquisition of mean beta weights in each participant for the ROI. Correlational analysis between the functional response in the dACC and the subjective craving, BDI and HAM-A scores were performed.

**Figure 2:**
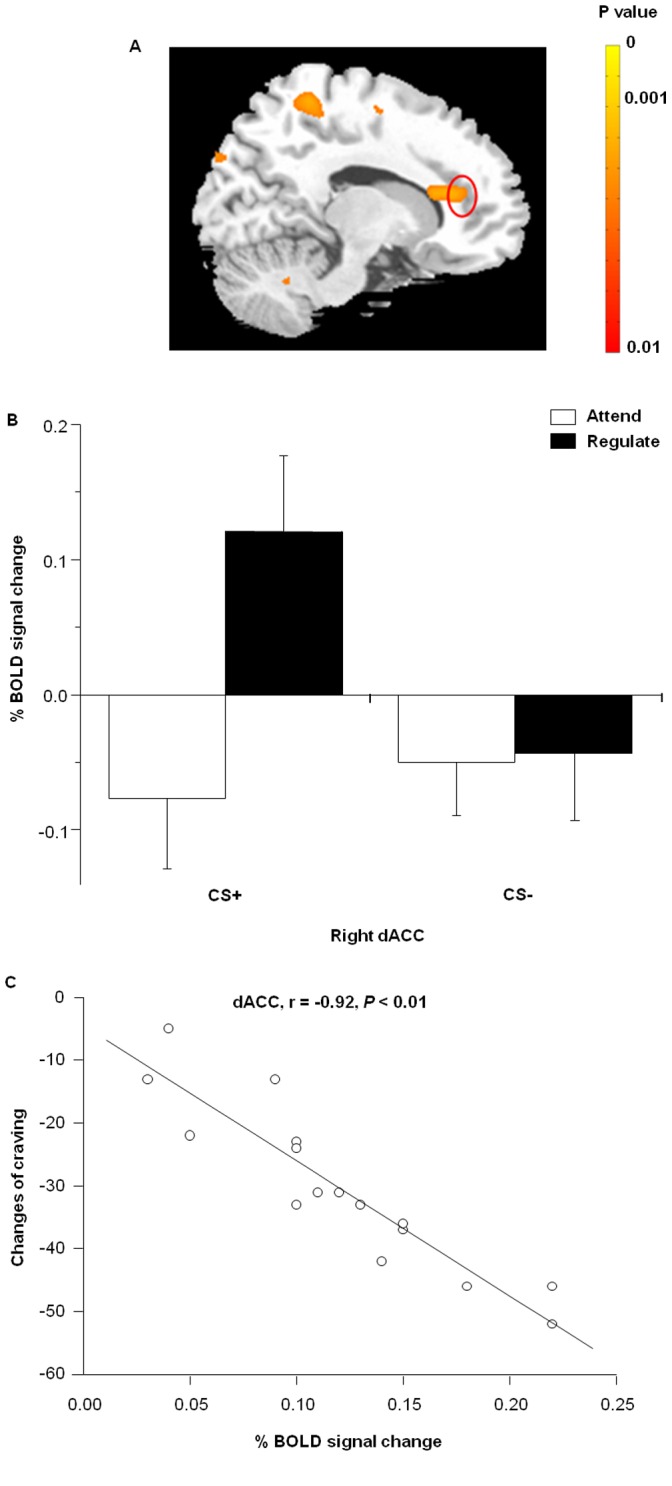
BOLD signals in the right dACC. (A) Activation of the right dACC reflected by the contrast between Regulate CS+ and Attend CS+ trials (cognitive reappraisal effects). (B) Mean beta weights from the right dACC showed an interaction of type of instruction and type of CS. (C) Craving significantly correlated with the differential dACC response to the Attend CS+ trials compared with the Regulate CS+ trials (r = −0.92, p<0.01; Fig. 2C). Craving scores during the cognitive strategies were calculated by subtracting the craving during Regulate CS+ trials from craving during Attend CS+ trials for each participant.

## Results

### Subjective Ratings

Craving ratings were compared using a repeated-measures ANOVA for type of instruction (Attend, Regulate) and type of stimulus (CS+, CS–) as within-subject factors (Fig. 1B). Craving showed a significant main effect of type of instruction (F_1,15_ = 59.19, p<0.001) and stimulus (F_1,15_ = 40.53, p<0.001) and a significant interaction between the two factors (F_1,15_ = 36.67, p<0.001). The results suggested that Attend and Regulate trials modulated the response to the CS in different ways. Using one-way repeated measures ANOVA, we compared the craving to the CS+ with CS– trials within instruction and the Regulate with Attention trials within stimuli. There was a significant difference between Attend CS+ and Regulate CS+ (F_1,15_ = 85.72, p<0.001). Compared Attend CS+ to Attend CS−, there was a significant difference (F_1,15_ = 20.74, p<0.001). The result indicated that using a reappraisal strategy was successful in decreasing the conditioned response to the CS+.

### Neuroimaging Results

To investigate the effects of cognitive reappraisal on cue-induced craving, we conducted a comparison between Attend CS+ and Regulate CS+ trials (Table 1). Within the subset of regions defined by this contrast, activation was observed in the prior ROI – dACC. To further investigate the response in the region, a 3 mm^3^ cube was drawn around the peak voxel of the target structure and applied to each individual participant to acquire percentage of signal change from a fixation baseline for four possible predictors (Attend CS+, Attend CS−, Regulate CS+ and Regulate CS−).

Right dACC (BA 24/32) activation occurred in response to the Regulate CS+ trials compared with the Attend CS+ trials (Fig. 2A). Using repeated-measures ANOVAs, we observed interaction between type of CSs and type of instruction in dACC (*F*_1,15_ = 52.63, *p*<0.05; Fig. 2B). *Post hoc* analysis revealed a differential response between Attend CS+ and Regulate CS+ trials (*t*_15_ = −0.4, *p* = 0.7), but not CS− trials (*t*_15_ = −9.97, *p* = 0.17). In addition, Craving significantly correlated with the differential dACC response to the Attend CS+ trials compared with the Regulate CS+ trials (r = −0.92, p<0.01; Fig. 2C). Craving scores during the cognitive strategies were calculated by subtracting the craving during Regulate CS+ trials from craving during Attend CS+ trials for each participant. RCS+ induced craving significantly correlated with (*r* = −0.92, *p*<0.01; Fig. 2C). The result suggests that the dACC activation contribute to the decrease of craving. There was no other significant correlation.

## Discussion

This investigation had two main findings. First, our reappraisal strategy successfully suppressed cue-induced craving in smokers. Second, we found negative correlations between RCS+ induced craving and BOLD signal changes in the right dACC. Consistent with the prior research, dACC activation plays an important role in examinations of brain function during cognitive reappraisal and cognitive modulation of emotion [4], [5], [6], [20].

Immediate reactions to emotionally evocative stimuli are not always helpful, and thus cognitive strategy is required to modify emotions toward specific goals [21]. The cognitive strategy of reappraisal significantly lowered subjects’ self-reported feelings of intensity, a finding that has been reported by several studies regarding both negative [6], [22], [23] and positive [11], [24], [25], [26] feelings. Our results suggested that cognitive reappraisal can be used to reduce their craving for cigarettes in smokers, which is consistent with the clinical findings that have demonstrated the efficacy of cognitive regulation in reducing craving and preventing relapse during smoking cessation [27].

Evidence showed that dACC play an important role in cognitive control of behavior [12]. Functional imaging of Go/No-Go and Stroop interference tasks that require subjects to restrain prepotent responses consistently show recruitment of dACC [28], [29], [30]. Activation in the dACC, which is implicated in conflict avoidance and attention control [28], [31], [32], may reflect the active direction of attention away from the hypersalient cigarette-related stimuli as a process that is different from automatic patterns of attention. Activity in the dACC might also be interpreted in terms of performance monitoring. One of these monitoring functions is the detection of response conflict [33]. In this study, response conflict caused by the reappraisal might have arisen from two competing tendencies: top-down signals vs. bottom-up reward signals. Top-down signals reflect the intention to reappraise the cigarette cues, whereas bottom-up reward signals promote the craving for the cigarette.

As drug addiction characterized that the saliency value of a drug and its related cues are enhanced, while the inhibitory control is weakened, setting up the stage for an unrestrained cycle which leads to compulsive drug-seeking without regard to its negative consequences [34], [35]. In the present study, smokers showed increased dACC activation in the cognitive reappraisal. The result may provide a biomarker of link between *cognitive control and drug cues.*

Our study has numerous limitations. First, similar to many other fMRI studies, ours was limited by a small sample size. Thus, although our confidence in these results is strengthened by the identification of *a priori* ROIs, these data must be considered preliminary. Future research with larger samples will be useful for increasing our confidence in this pattern of findings. Second, we restricted the sample only to males, although research has demonstrated that male and female smokers differ in their responses to smoking-related stimuli and nicotine administration [36], [37]. We sought to reduce the possibility of introducing such variance given the relatively small sample size. Whether our findings generalize to female smokers awaits direct investigation. Third, the paradigm may potentially measure craving *vs*. reward expectation. Previous research suggested that manipulations that generate expectations of cigarette smoking can increase cigarette craving in smokers [38] and may potentiate cue-induced cigarette craving [39]. Using the same paradigm, the presentation of cues previously paired with cigarettes can elicit craving compared with stimuli that predict the absence of cigarettes in smokers [40]. Fourth, the study design lacks a nonsmoking control group exposed to the same cues as the smokers. However, in prior work [41], nonsmokers demonstrated neither cigarette craving nor changes in mood/anxiety associated with the presentation of cigarette-related cues. Additionally, in our pilot study, the cigarette cue did not induce craving in nonsmoking control subjects. However, in this study, the primary analysis was the investigation of cigarette cue exposure under Regulate and Attend conditions. The use of nonsmoking control subjects would not be expected to help the central interpretation of the study.

Altered sensitivity to the incentive salience of drug cues has been proposed as the neural basis of pathological “wanting” (Volkow and Wise, 2005). The dACC, which might mediate the neural circuit involved in the craving response, provides a target for top-down cognitive interventions that may be therapeutically beneficial. This strategy may help addicted individuals avoid the anticipated situations where they are exposed to conditioned cues. Our findings suggest that smokers can use self-instruction to inhibit the craving response when they are exposed to cigarette cues. In fact, addicts are inevitably exposed to drug cues in their daily lives; therefore, the acquisition of the ability to exert cognitive control may decrease relapse rates.

## References

[1] Shiffman S, Balabanis MH, Gwaltney CJ, Paty JA, Gnys M, et al (2007) Prediction of lapse from associations between smoking and situational antecedents assessed by ecological momentary assessment. Drug Alcohol Depend 91: 159–168. 17628353 10.1016/j.drugalcdep.2007.05.017 PMC2244586

[2] Engelmann JM, Versace F, Robinson JD, Minnix JA, Lam CY, et al (2012) Neural substrates of smoking cue reactivity: a meta-analysis of fMRI studies. Neuroimage 60: 252–262. 22206965 10.1016/j.neuroimage.2011.12.024 PMC3288122

[3] Giuliani NR, Drabant EM, Bhatnagar R, Gross JJ (2011) Emotion regulation and brain plasticity: expressive suppression use predicts anterior insula volume. Neuroimage 58: 10–15. 21704173 10.1016/j.neuroimage.2011.06.028 PMC3161031

[4] Kalisch R, Wiech K, Critchley HD, Dolan RJ (2006) Levels of appraisal: a medial prefrontal role in high-level appraisal of emotional material. Neuroimage 30: 1458–1466. 16388969 10.1016/j.neuroimage.2005.11.011

[5] Ray RD, Ochsner KN, Cooper JC, Robertson ER, Gabrieli JD, et al (2005) Individual differences in trait rumination and the neural systems supporting cognitive reappraisal. Cogn Affect Behav Neurosci 5: 156–168. 16180622 10.3758/cabn.5.2.156

[6] Ochsner KN, Ray RD, Cooper JC, Robertson ER, Chopra S, et al (2004) For better or for worse: neural systems supporting the cognitive down- and up-regulation of negative emotion. Neuroimage 23: 483–499. 15488398 10.1016/j.neuroimage.2004.06.030

[7] Phillips ML, Drevets WC, Rauch SL, Lane R (2003) Neurobiology of emotion perception II: Implications for major psychiatric disorders. Biol Psychiatry 54: 515–528. 12946880 10.1016/s0006-3223(03)00171-9

[8] Paus T, Koski L, Caramanos Z, Westbury C (1998) Regional differences in the effects of task difficulty and motor output on blood flow response in the human anterior cingulate cortex: a review of 107 PET activation studies. Neuroreport 9: R37–47. 9674567 10.1097/00001756-199806220-00001

[9] Ma N, Liu Y, Fu XM, Li N, Wang CX, et al (2011) Abnormal brain default-mode network functional connectivity in drug addicts. PLoS One 6: e16560. 21298074 10.1371/journal.pone.0016560 PMC3027699

[10] Ma N, Liu Y, Li N, Wang CX, Zhang H, et al (2010) Addiction related alteration in resting-state brain connectivity. Neuroimage 49: 738–744. 19703568 10.1016/j.neuroimage.2009.08.037 PMC2764798

[11] Delgado MR, Gillis MM, Phelps EA (2008) Regulating the expectation of reward via cognitive strategies. Nat Neurosci 11: 880–881. 18587392 10.1038/nn.2141 PMC3077128

[12] Miller EK, Cohen JD (2001) An integrative theory of prefrontal cortex function. Annu Rev Neurosci 24: 167–202. 11283309 10.1146/annurev.neuro.24.1.167

[13] Domino EF, Ni L (2002) Clinical phenotyping strategies in selection of tobacco smokers for future genotyping studies. Prog Neuropsychopharmacol Biol Psychiatry 26: 1071–1078. 12452528 10.1016/s0278-5846(02)00224-5

[14] Hamilton M (1959) The assessment of anxiety states by rating. Br J Med Psychol 32: 50–55. 13638508 10.1111/j.2044-8341.1959.tb00467.x

[15] Beck AT, Steer RA, Ball R, Ranieri W (1996) Comparison of Beck Depression Inventories -IA and -II in psychiatric outpatients. J Pers Assess 67: 588–597. 8991972 10.1207/s15327752jpa6703_13

[16] Fagerstrom KO, Schneider NG (1989) Measuring nicotine dependence: a review of the Fagerstrom Tolerance Questionnaire. J Behav Med 12: 159–182. 2668531 10.1007/BF00846549

[17] Cox LS, Tiffany ST, Christen AG (2001) Evaluation of the brief questionnaire of smoking urges (QSU-brief) in laboratory and clinical settings. Nicotine Tob Res 3: 7–16. 11260806 10.1080/14622200020032051

[18] Woolrich MW, Ripley BD, Brady M, Smith SM (2001) Temporal autocorrelation in univariate linear modeling of FMRI data. Neuroimage 14: 1370–1386. 11707093 10.1006/nimg.2001.0931

[19] Giuliani NR, Drabant EM, Gross JJ (2011) Anterior cingulate cortex volume and emotion regulation: is bigger better? Biol Psychol 86: 379–382. 21138751 10.1016/j.biopsycho.2010.11.010 PMC3051027

[20] Pessoa L, Kastner S, Ungerleider LG (2002) Attentional control of the processing of neural and emotional stimuli. Brain Res Cogn Brain Res 15: 31–45. 12433381 10.1016/s0926-6410(02)00214-8

[21] Phan KL, Fitzgerald DA, Nathan PJ, Moore GJ, Uhde TW, et al (2005) Neural substrates for voluntary suppression of negative affect: a functional magnetic resonance imaging study. Biol Psychiatry 57: 210–219. 15691521 10.1016/j.biopsych.2004.10.030

[22] Kalisch R, Wiech K, Critchley HD, Seymour B, O’Doherty JP, et al (2005) Anxiety reduction through detachment: subjective, physiological, and neural effects. J Cogn Neurosci 17: 874–883. 15969906 10.1162/0898929054021184

[23] Eippert F, Veit R, Weiskopf N, Erb M, Birbaumer N, et al (2007) Regulation of emotional responses elicited by threat-related stimuli. Hum Brain Mapp 28: 409–423. 17133391 10.1002/hbm.20291 PMC6871321

[24] Beauregard M, Levesque J, Bourgouin P (2001) Neural correlates of conscious self-regulation of emotion. J Neurosci 21: RC165. 11549754 10.1523/JNEUROSCI.21-18-j0001.2001 PMC6763007

[25] Kim SH, Hamann S (2007) Neural correlates of positive and negative emotion regulation. J Cogn Neurosci 19: 776–798. 17488204 10.1162/jocn.2007.19.5.776

[26] Staudinger MR, Erk S, Abler B, Walter H (2009) Cognitive reappraisal modulates expected value and prediction error encoding in the ventral striatum. Neuroimage 47: 713–721. 19442745 10.1016/j.neuroimage.2009.04.095

[27] O’Connell KA, Hosein VL, Schwartz JE, Leibowitz RQ (2007) How does coping help people resist lapses during smoking cessation. Health Psychology 26: 77–84. 17209700 10.1037/0278-6133.26.1.77

[28] Braver TS, Barch DM, Gray JR, Molfese DL, Snyder A (2001) Anterior cingulate cortex and response conflict: effects of frequency, inhibition and errors. Cereb Cortex 11: 825–836. 11532888 10.1093/cercor/11.9.825

[29] Konishi S, Nakajima K, Uchida I, Kikyo H, Kameyama M, et al (1999) Common inhibitory mechanism in human inferior prefrontal cortex revealed by event-related functional MRI. Brain 122 (Pt 5): 981–991. 10.1093/brain/122.5.981 10355680

[30] Garavan H, Ross TJ, Murphy K, Roche RA, Stein EA (2002) Dissociable executive functions in the dynamic control of behavior: inhibition, error detection, and correction. Neuroimage 17: 1820–1829. 12498755 10.1006/nimg.2002.1326

[31] Barch DM, Braver TS, Akbudak E, Conturo T, Ollinger J, et al (2001) Anterior cingulate cortex and response conflict: effects of response modality and processing domain. Cereb Cortex 11: 837–848. 11532889 10.1093/cercor/11.9.837

[32] Liu X, Banich MT, Jacobson BL, Tanabe JL (2004) Common and distinct neural substrates of attentional control in an integrated Simon and spatial Stroop task as assessed by event-related fMRI. Neuroimage 22: 1097–1106. 15219581 10.1016/j.neuroimage.2004.02.033

[33] Dreher JC, Grafman J (2003) Dissociating the roles of the rostral anterior cingulate and the lateral prefrontal cortices in performing two tasks simultaneously or successively. Cereb Cortex 13: 329–339. 12631562 10.1093/cercor/13.4.329

[34] Baler RD, Volkow ND (2006) Drug addiction: the neurobiology of disrupted self-control. Trends Mol Med 12: 559–566. 17070107 10.1016/j.molmed.2006.10.005

[35] Goldstein RZ, Volkow ND (2002) Drug addiction and its underlying neurobiological basis: neuroimaging evidence for the involvement of the frontal cortex. Am J Psychiatry 159: 1642–1652. 12359667 10.1176/appi.ajp.159.10.1642 PMC1201373

[36] Heishman SJ, Lee DC, Taylor RC, Singleton EG (2010) Prolonged duration of craving, mood, and autonomic responses elicited by cues and imagery in smokers: Effects of tobacco deprivation and sex. Exp Clin Psychopharmacol 18: 245–256. 20545389 10.1037/a0019401 PMC2896221

[37] Perkins KA, Gerlach D, Vender J, Grobe J, Meeker J, et al (2001) Sex differences in the subjective and reinforcing effects of visual and olfactory cigarette smoke stimuli. Nicotine Tob Res 3: 141–150. 11403728 10.1080/14622200110043059

[38] Juliano LM, Brandon TH (1998) Reactivity to instructed smoking availability and environmental cues: evidence with urge and reaction time. Exp Clin Psychopharmacol 6: 45–53. 9526145 10.1037//1064-1297.6.1.45

[39] Droungas A, Ehrman RN, Childress AR, O’Brien CP (1995) Effect of smoking cues and cigarette availability on craving and smoking behavior. Addict Behav 20: 657–673. 8712062 10.1016/0306-4603(95)00029-c

[40] Field M, Duka T (2001) Smoking expectancy mediates the conditioned responses to arbitrary smoking cues. Behav Pharmacol 12: 183–194. 11485055 10.1097/00008877-200105000-00004

[41] Brody AL, Mandelkern MA, London ED, Childress AR, Lee GS, et al (2002) Brain metabolic changes during cigarette craving. Arch Gen Psychiatry 59: 1162–1172. 12470133 10.1001/archpsyc.59.12.1162

